# Activating Mutation of SHP2 Establishes a Tumorigenic Phonotype Through Cell-Autonomous and Non-Cell-Autonomous Mechanisms

**DOI:** 10.3389/fcell.2021.630712

**Published:** 2021-03-11

**Authors:** Lei Dong, Da Han, Xinyi Meng, Mengchuan Xu, Chuwen Zheng, Qin Xia

**Affiliations:** ^1^School of Life Sciences, Beijing Institute of Technology, Beijing, China; ^2^School of Biological Sciences, Georgia Institute of Technology, Atlanta, GA, United States

**Keywords:** SHP2 mutation, tumor, cell-autonomous/non-cell autonomous mechanisms, SHP2 inhibition, tumor microenvironment

## Abstract

Gain-of-function mutation of SHP2 is a central regulator in tumorigenesis and cancer progression through cell-autonomous mechanisms. Activating mutation of SHP2 in microenvironment was identified to promote cancerous transformation of hematopoietic stem cell in non-autonomous mechanisms. It is interesting to see whether therapies directed against SHP2 in tumor or microenvironmental cells augment antitumor efficacy. In this review, we summarized different types of gain-of-function SHP2 mutations from a human disease. In general, gain-of-function mutations destroy the auto-inhibition state from wild-type SHP2, leading to consistency activation of SHP2. We illustrated how somatic or germline mutation of SHP2 plays an oncogenic role in tumorigenesis, stemness maintenance, invasion, etc. Moreover, the small-molecule SHP2 inhibitors are considered as a potential strategy for enhancing the efficacy of antitumor immunotherapy and chemotherapy. We also discussed the interconnection between phase separation and activating mutation of SHP2 in drug resistance of antitumor therapy.

## Introduction

Protein tyrosine phosphatases (PTPs) are widely expressed in most tissues. They play a regulatory role in various cell signaling events, such as mitogenic activation, metabolic control, transcription regulation, and cell migration. Src homology region 2 protein tyrosine phosphatase 2 (SHP2), encoded by *PTPN11*, is the first reported non-receptor protein oncogenic tyrosine phosphatase and required for the survival, proliferation, and differentiation of multiple cell types (Yang et al., 2013). Studies reported that germline mutations in the *PTPN11* gene contribute to Noonan syndrome (NS) (Niemeyer, 2018; Pierpont and Digilio, 2018; Bellio et al., 2019), which is a multisystem developmental disorder disease characterized by short stature, chest deformity, webbed neck, bleeding diatheses, cardiac defects, and mental retardation (Grossmann et al., 2010; Roberts et al., 2013; Liu et al., 2020). Patients with NS tend to develop juvenile myelomonocytic leukemia (JMML)-like myeloproliferative neoplasm (MPN) (Strullu et al., 2014). Hyperactive Ras signaling is the main driving event caused by somatic mutations in *KRAS*, *NRAS*, or *PTPN11* in about 50% of JMML patients (Tartaglia et al., 2004; Lipka et al., 2017). Mutations in *NF1*, *NRAS*, *KRAS*, *CBL*, and *PTPN11* account for diagnosis in 85% of JMML patients (Stieglitz et al., 2015). Germline mutation of *PTPN11* is found in 50% of the patients with NS (Dong et al., 2016). Somatic *PTPN11* mutations are also associated with multiple types of human malignancies, such as leukemia and other solid tumors (Yang et al., 2013). According to previous reports, *PTPN11* mutations affect disease progression by unblocking PTP activity and enhancement of the catalytic activity via disrupting the auto-inhibition status or regulating the substrate binding ability of the catalytic pocket (Guo et al., 2017). SHP2 is proved to promote tumor proliferation, invasion, metastasis, and chemotherapeutic resistance (Zhang et al., 2015).

Gain-of-function (GOF) mutation SHP2 promotes tumor progression in cell-autonomous and non-autonomous mechanisms. SHP2 plays a central and indispensable role in hematopoiesis and leukemogenesis via its complex involvement with cellular signaling pathways (Pandey et al., 2017). Furthermore, activating mutations SHP2 in the bone marrow microenvironment, but not in the tumor cells, also promote childhood MPN development and progression through detrimental effects on hematopoietic stem cells (HSCs) in non-autonomous mechanism (Dong et al., 2016). Thus, a comprehensive understanding of how SHP2 contributes to oncogenesis will provide novel insights into pathogenesis.

It was of great interest to discover small-molecule SHP2 inhibitors as a potential cancer therapeutic target in recent years. The study in SHP2 inhibition did not make a breakthrough until the discovery of inhibitors that occupied allosteric sites of SHP2 (Chen et al., 2016; Shen et al., 2020). This novel discovery shed light on efficient SHP2 inhibitors (Garcia Fortanet et al., 2016). Targeting these non-conserved allosteric sites tends to improve drug selectivity. Consequently, several other allosteric drugs were continuously discovered with higher expectation for cell permeability, oral availability, etc. (Chen et al., 2016; Shen et al., 2020). Currently, a few clinical trials of SHP2 allosteric inhibitors showed remarkable antitumor benefits (Liu et al., 2020).

In this review, we summarized the structural change and functional regulation of oncogenic SHP2 mutations. We discussed how SHP2 affects tumor progressions in cell-autonomous and non-autonomous mechanisms. Since SHP2 is considered as a novel antitumor target, we also summarized currently used SHP2 inhibitors as well as their potentials in the application of cancer treatment.

## The Structural Conformation Changes and Functional Regulation of Oncogenic SHP2

SHP2 consists of one PTP catalytic domain that locates at the C-terminal region, two tandem C-SH2 and N-SH2 domains, and a C-terminal tail with tyrosyl phosphorylation sites (Feng et al., 1993). Human SHP2 encodes 593 amino acids, among which the N-SH2 domain locates at 3–104, C-SH2 domain locates at 112–216, the PTP domain locates at 221–524, and the C-terminal locates at 525–593. The N-SH2 domain has two non-overlapping ligand binding sites to regulate its de-phosphorylated activity. The C-SH2 domain provides binding energy and specificity (Zhang et al., 2015). The PTP domain contains the catalytic structures, such as the P ring (Yu et al., 2013), to de-phosphorylate substrates.

**FIGURE 1:**
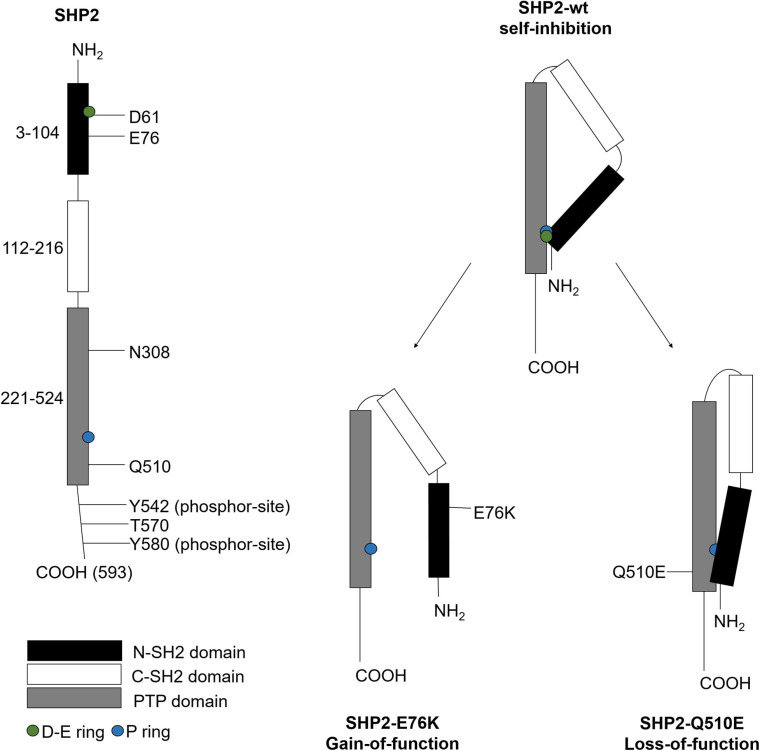
The structure and function regulation of SHP2. SHP2 contains 593 amino acids, including various important active regulatory sites. When these sites are mutated, SHP2 gains or loses function. The D-E ring and P ring on the three-dimensional structure of SHP2 play an important role in regulating catalytic activity. When SHP2 is in self-inhibition state, the P ring on the PTP domain will cover the D-E ring on the N-SH2 domain. SHP2 is activated by gain-of-function mutations, such as E76K, or inactivated by loss-of-function mutations, such as Q510E.

SHP2 activity is regulated by conformational switch that N-SH2 binds to PTP to block or binds to phosphorylated proteins to unblock its phosphatase activity (Zhang et al., 2015). SHP2 mainly exists in a closed self-inhibitory conformation (Zhang et al., 2020). In the inactive state, the D-E ring of the N-SH2 domain is inserted into the PTP domain to block the phosphatase activity site (Rehman et al., 2019). Studies reported that the stimulation of growth factor receptor [e.g., epidermal growth factor receptor (EGFR)] or the interaction between the N-SH2 domain with phosphorylated tyrosine residues of scaffold proteins led to the dissociation of N-SH2 with the PTP domain; thus, the active region of PTP will be exposed and SHP2 is activated (Liu et al., 2016). The structure and function regulation of SHP2 is shown in Figure 1. In addition, SHP2 is activated via the phosphorylation on two tyrosine residues (Y542 and Y580) within the C-terminal region (Voena et al., 2007).

Gain-of-function mutations of SHP2 affect the interaction between N-SH2 and PTP (Tajan et al., 2015). GOF mutations mainly occur in the N-SH2 or PTP domains of cancer patients. These mutations lead to partial or complete dissociation of the binding domain of N-SH2 and PTP, and enhancement of phosphatase activity (Pannone et al., 2017). For example, the N-SH2 domain of *PTPN11*^*E*76*K*/+^ mutant leads to consistently expose the catalytic site of the PTP domain (LaRochelle et al., 2018).

Protein tyrosine phosphatase is the catalytic domain that mediates a variety of cellular signaling processes including cell growth, differentiation, mitotic cycle, and oncogenic transformation. Several GOF mutations or loss-of-function (LOF) mutations of SHP2 are reported to be associated with cancer progression and other diseases (Table 1). Multiple researches reported that GOF mutations SHP2^*D*61*G*^ and SHP2^*E*76*K*^ are related to MPN (Xu et al., 2010), NS (De Rocca Serra-Nédélec et al., 2012), or JMML (Yang et al., 2008) through activation of Ras/Erk signaling *in vivo* and *in vitro*. SHP2^*E*76*K*^ promotes tumorigenesis of colorectal cancer (CAC) and induces epithelial-to-mesenchymal transition (EMT) through the Wnt/β-catenin signaling pathway (Schneeberger et al., 2014). Studies reported that SHP2 is required for the growth of KRAS-mutant non-small-cell lung cancers (NSCLCs), and its inhibition leads to a potential antitumor therapy (Mainardi et al., 2018). More details are shown in Table 1.

**TABLE 1:** Gain/loss-of-function mutations of SHP2.

Mutants	Disease	Influences and mechanisms	Function	References
D61G	/	SHP2^*D61G*^ promotes U251 proliferation and inhibits apoptosis	GOF	Zhao et al., 2017
D61G	/	SHP2^*D61G*^ enhances the production of ROS, leading to abnormal proliferation of bone marrow	GOF	Xu et al., 2013
D61G	JMML	JMML is a myeloproliferative neoplasm (MPN) of childhood with a poor prognosis. SHP2^*D61G*^ promotes abnormal activation of hematopoietic stem cells and leads to JMML	GOF	Xu et al., 2010
D61G	NS	NS is a multisystem developmental disease. Patients with NS tend to develop JMML. SHP2^*D61G*^ promotes hyperactivation of Ras/Erk1/2 to inhibit GH-induced IGF-1 release, leading to growth retardation and NS	GOF	De Rocca Serra-Nédélec et al., 2012
D61G	Breast cancer	SHP2^*D61G*^ activates GAB1/Ras/Erk axis to promote breast cancer invasion and migration	GOF	Hu et al., 2016
D61Y	JMML	SHP2^*D61Y*^ promotes the cell cycle development and survival of hematopoietic progenitor cells and further leads to JMML	GOF	Yang et al., 2008
E76K	JMML	SHP2^*E76K*^ promotes the cell cycle development and survival of hematopoietic progenitor cells	GOF	Yang et al., 2008
E76K	GBM	GBM is the most aggressive and common form of brain malignancy in adults. SHP2^*E76K*^ activates the Erk/CREB pathway to promote GBM cell proliferation, metastasis, and tumor growth	GOF	Yang et al., 2019
E76K	/	SHP2^*E76K*^ leads to mitotic abnormalities	GOF	Liu et al., 2016
E76K	/	SHP2^*E76K*^ promotes lung tumor development in transgenic mice	GOF	Schneeberger et al., 2014
E76K	/	SHP2^*E76K*^ enhances the production of ROS, leading to abnormal proliferation of bone marrow	GOF	Xu et al., 2013
E76K	/	SHP2^*E76K*^ has a non-pedigree-specific effect on hematopoietic malignant transformation and leads to acute leukemia in every stage of hematopoiesis	GOF	Xu et al., 2011
E76K	CRC	The mutation rate of SHP2 is the highest in CRC. SHP2^*E76K*^ promotes tumorigenesis and induces EMT through the Wnt/β-catenin signaling pathway	GOF	Zhang et al., 2018
E76K	Hydrocephalus	SHP2^*E76K*^ promotes the pathogenesis of hydrocephalus in mice by inhibition of STAT3 and enhancement of Erk/Akt activity. SHP2^*C459S*^ suppresses this pathogenic effect	GOF	Zheng et al., 2018
E76Q	/	Phosphatase activity of SHP2^*E76Q*^ was enhanced	GOF	Rehman et al., 2019
T507K	/	SHP2^*T507K*^ dephosphorylates Sprouty1 to hyperactive Ras signaling pathway	GOF	Zhang et al., 2020
Q506P	/	Phosphatase activity of SHP2^*Q506P*^ is reduced	LOF	Noda et al., 2016
Q510E	HCM	Dysregulation of mTOR signal pathway mediated by SHP2^*Q510E*^ causes HCM, which is a common inherited cardiovascular disease	LOF	Schramm et al., 2012
Q510E	HCM	SHP2^*Q510E*^ mutation reduces cardiac cell differentiation and promotes cardiac hypertrophy by disabling Akt/GSK-3/β-catenin signaling pathway	LOF	Ishida et al., 2011
T468M	/	Phosphatase activity of SHP2^*T468M*^ is reduced	LOF	Noda et al., 2016
Y279C	/	Phosphatase activity of SHP2^*Y279C*^ is reduced	LOF	Xu et al., 2010

*NS, Noonan syndrome; JMML, juvenile myelomonocytic leukemia; CRC, colorectal cancer; GBM, glioblastoma multiforme; HCM, hypertrophic cardiomyopathy; LOF, loss of function; GOF, gain of function; EMT, epithelial-to-mesenchymal transition.*

A recent study reported that multivalent electrostatic interaction among the PTP domains leads to liquid–liquid phase separation (LLPS) of mutated SHP2. It recruits wild-type SHP2 and hyperactivates the phosphatase catalytic activity of SHP2, which further leads to hyperactivation of mitogen-activated protein kinase (MAPK) signaling pathway (Zhu et al., 2020). It also reported that SHP2 mutants in LEOPARD syndrome induce robust phase transition to liquid-like droplets in cells, which recruit and activate wild-type SHP2 to promote extracellular signal-regulated kinase1/2 (Erk1/2) activation (Zhu et al., 2020). It suggests that disease-associated SHP2 mutations promote GOF LLPS and consequently lead to overactivation of wild-type SHP2. The formation of LLPS is a mechanism of SHP2 activation and a potential contributor to developmental diseases and cancers.

## Gain-of-Function SHP2 Promotes Tumor Progression in Cell-Autonomous or Non-Autonomous Mechanisms

The oncogenic SHP2 promotes cancer progression at a cellular level through two mechanisms. On the one hand, malignant proliferation results from tumor cell-autonomous oncogenic SHP2 mutations. Studies have reported that SHP2^*D*61*Y*^ causes fatal myeloproliferative disorder via cell-autonomous effects on multiple stages of hematopoiesis (Chan et al., 2009). On the other hand, SHP2 in tumor microenvironment cells, such as mesenchymal stem cells (MSCs) and/or immunological cells, is responsible for tumor progression (Dong et al., 2016).

### SHP2 Mutations Promote Tumor Progression in Cell-Autonomous Mechanism

It has been established that oncogenic alterations in the Ras/Raf/MEK/Erk pathway drive the neoplasia of multiple cancer types. SHP2 is expressed in multiple types of cells and regulates cell survival and proliferation through activation of the Ras/Erk signaling pathway (Chan et al., 2008; Bondeson, 2017). SHP2 negatively regulates the cytokine receptor-mediated JAK-STAT signaling pathway (Xu and Qu, 2008). Some studies also reported that SHP2^*E*76*K*^ in the glioblastoma multiforme (GBM) cells promotes the malignant behavior of tumor cells through the Erk/cAMP responsive element binding protein (CREB) signaling pathway (Yang et al., 2019). These evidences indicated that activated SHP2 in tumor cells established oncogenic signaling pathways to promote tumor progression.

SHP2 established proliferative signaling pathways to promote tumorigenesis. Studies showed that SHP2^*E*76*K*^ activates Erk and Src to promote the occurrence of lung tumors (Schneeberger et al., 2014). SHP2 dephosphorylates Ras to increase the association between Ras and Raf, thus activating the proliferation-promoting Ras/Erk/MAPK signaling pathway. Overexpression of SHP2 activates Erk/Akt signaling pathways and further leads to tumorigenesis of breast cancer (Hu et al., 2014). SHP2 activity is elevated by pathological analysis of astrocytes isolated from GBM. Patient-derived GBM specimens exhibit hyperactive Ras, while inhibition of SHP2 decelerates the progression of low-grade astrocytoma to GBM in a spontaneous transgenic glioma mouse model (Bunda et al., 2015). The observation that conditional knockout of SHP2 in the ErbB2 transgenic mice prevents tumorigenesis by blocking the expression of the ErbB2 indicates that SHP2 induces tumorigenesis through regulating the expression of oncogene (Zhao H. et al., 2019). Studies also revealed that SHP2 affects proliferation and tumorigenicity of glioblastoma stem cells (GSCs) through regulating the expression of transcription factor SOX2 (Roccograndi et al., 2017).

Oncogenic SHP2 promotes tumor progression. It has been demonstrated that targeting both Ras and its upstream or downstream proteins has no cancer-suppressing effect in Ras-mutant cancer (Bernards, 2012). However, studies revealed that SHP2 inhibition in KRAS-mutant NSCLCs *in vivo* under growth factor-limiting conditions triggers senescence response (Mainardi et al., 2018). Furthermore, genetic deletion of *PTPN11* or inhibition of SHP2 in KRAS-mutant-driven tumors delays tumor progression (Ruess et al., 2018). Another study pointed out that SHP2 small molecular allosteric inhibitor RMC-4550 decreases oncogenic-related Ras/Raf/MEK/Erk signaling to impair the growth of cancer-bearing Ras-GTP-dependent oncogenic BRAF mutation, NF1 loss, or nucleotide-cycling oncogenic Ras (Nichols et al., 2018). Wang et al. showed that SHP2^*E*76*K*^ and SHP2^D61G^ induce cytokine allergy of hematopoietic cells by enhancing the production of reactive oxygen species (ROS). They interact with a new substrate in the mitochondria to increase the aerobic metabolism of the mitochondria and drive the development of myeloproliferative diseases and malignant leukemia (Xu et al., 2013).

Additionally, SHP2 promotes tumor metastasis. SHP2 decreases the phosphorylation of PAR3 (partitioning-defective 3) to impair the formation of polarity-regulating protein complex, resulting in a disrupted cell polarity, dysregulated cell–cell junctions, and increased EMT, which is one of the essential steps for prostate cancer metastasis (Zhang et al., 2016). Other studies demonstrated that SHP2 overexpression enhances ovarian tumor invasion by activating the PI3K/Akt axis (Hu et al., 2017). SHP2 knockdown inhibits cell migration in the HeLa and SiHa cervical cancer cell lines, while SHP2 overexpression has the opposite effects. This study further pointed out that the tumor-promoting effect of SHP2 is partially related to Akt signaling (Cao et al., 2019). Other studies reported that SHP2^*E*76*K*^ promotes GBM tumor metastasis via the activation of Erk/CREB axis (Yang et al., 2019).

**FIGURE 2:**
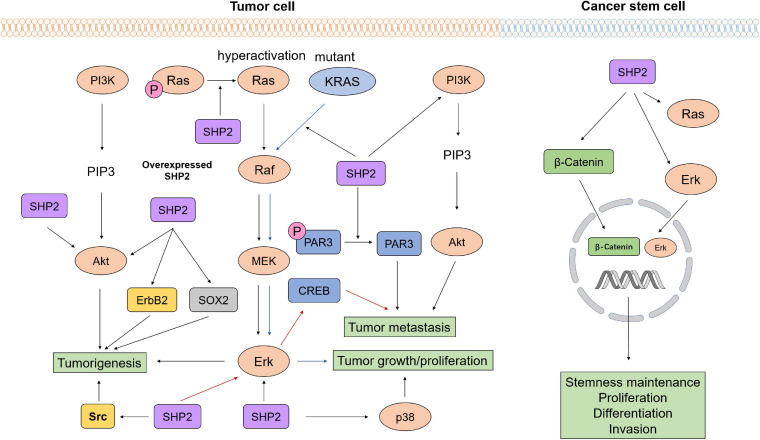
The oncogenic function of tumor cell-autonomous SHP2 gain-of-function mutations. As a signaling hub, SHP2 affects various tumor developmental stages by regulating multiple signaling pathways, such as PI3K/Akt, Ras/Raf/MEK/Erk signaling. SHP2 promotes proliferation, differentiation, and invasion and maintains stemness of cancer stem cells via activation of β-catenin and Ras/Erk signaling.

Gain-of-function mutations of SHP2 in cancer stem cells (CSCs) promote cell expansion, proliferation, and stemness maintenance and are responsible for drug resistance. Studies reported that activated SHP2 in CSCs promotes liver CSC expansion by activating β-catenin signaling (Xiang et al., 2017). Treatment of NSCLC by tyrosine kinase inhibitor (TKI) failed because SHP2 induces the stemness of KRAS-mutant NSCLCs. The inhibition of SHP2 attenuates the enhanced stemness (Jiang et al., 2019), suggesting the important role of tumor cell-autonomous SHP2 in stemness maintenance of CSCs. Other studies revealed that SHP2 catalytic activity is required for proliferation and tumorigenic transformation of GSCs (Roccograndi et al., 2017). A recent study revealed that *PTPN11*^*G*226*A*^ mutation is essential in hematopoietic differentiation of JMML-derived induced pluripotent stem cells (iPSCs), suggesting the significant role of SHP2 in regulating stem cell bioactivity (Shigemura et al., 2019). The oncogenic function of tumor cell-autonomous SHP2 is shown in Figure 2.

### SHP2 in Tumor Microenvironment Affects Tumor Progression via Non-autonomous Mechanism

Cre/LoxP system is applied to elucidate the specific role of non-autonomous SHP2 mutations in cancer. Some studies examined the detailed pathogenesis of metachondromatosis by deleting *PTPN11* specifically in monocytes, macrophages, and osteoclasts (lysozyme M-Cre; LysM-Cre) or in cathepsin K (Ctsk)-expressing cells using *PTPN11*^*flox/flox*^ and Cre recombinase transgenic mice (Yang et al., 2013). *PTPN11* deletion in CD4^+^ cells driven by CD4 Cre recombinase demonstrated that although the ablation of SHP2 does not affect T cell development and functions, it causes cartilage tumors in a T cell-independent manner (Miah et al., 2017). More importantly, a previous study on the leukemogenic effect of SHP2 mutation in bone marrow microenvironment generated *PTPN11*^*E*76*K/+*^/Nestin-Cre^+^ transgenic mice with a neo cassette and a stop codon inserted ahead of *PTPN11*^*E*76*K*^, and thus, the SHP2^*E*76*K*^ expressed with the deletion of neo cassette by Cre DNA recombinase (Dong et al., 2016). Ding et al. (2012) studied the cellular sources of Scf (stem cell factor) that affects HSC frequency and function by conditionally deleting Scf from hematopoietic cells, osteoblasts, nestin-cre- or nestin-creER-expressing cells, endothelial cells, or leptin receptor (Lepr)-expressing perivascular stromal cells. They found that HSCs were depleted when Scf was conditionally deleted in perivascular cells; thus, HSCs were proved to reside in a perivascular niche where they remained undifferentiated. Studying the oncogenic effects of SHP2 mutations in different cell populations in the tumor microenvironment could follow similar methods. In general, the use of transgenic mice combined with the Cre/LoxP system is a reliable approach for studying the role of SHP2 in diseases, especially in tumors.

#### SHP2 Mutations in Bone Marrow Microenvironment Promotes Leukemogenesis

**FIGURE 3:**
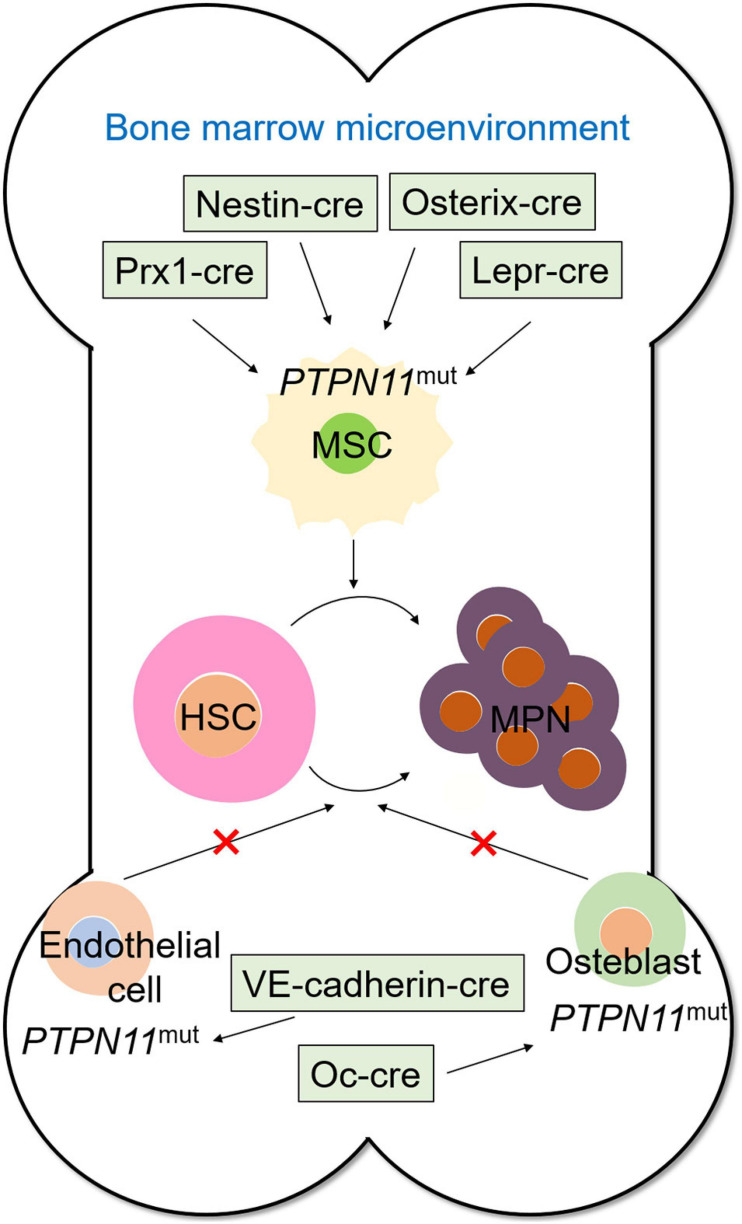
SHP2 mutation in bone marrow microenvironment promotes myeloproliferative neoplasm. *PTPN11* mutation induced by Prx1-cre, Nestin-cre, Osterix-cre, and Lepr-cre in mesenchymal stem cells promotes tumorigenicity of hematopoietic stem cells, whereas *PTPN11* mutation induced by VE-Cadherin-cre in endothelial cells and Oc-cre in osteoblasts shows no myeloproliferative neoplasm formation-promoting effect.

SHP2 mutations promote the leukemogenesis of HSCs in non-cell-autonomous mechanisms. HSCs reside in distinct bone marrow niches defined by the surrounding stromal cells and the regulatory molecules they produce (Greenbaum et al., 2013), wherein the transduction signaling generated by surrounding microenvironment cells affects the self-renewal, proliferation of HSCs, and MPN formation. Studies have demonstrated that *PTPN11* mutation in the bone marrow microenvironment, such as MSCs, promotes the development and progression of childhood MPN through the profound negative effects on HSCs (Dong et al., 2016), which demonstrated that not only tumor cell-autonomous SHP2 but also mutations in microenvironment cells leads to tumorigenesis. This study clarified the mechanism of leukemia recurrence. Using specifically expressed cre, such as Prx1-cre (Greenbaum et al., 2013), nestin-cre (Méndez-Ferrer et al., 2010), Lepr-cre (Ding et al., 2012), and Osterix-cre (Tang et al., 2016), to induce *PTPN11* mutations in bone marrow mesenchymal cells leads to MPN, but SHP2 mutations in endothelial cells (VE-Cadherin-cre) and osteoblasts (Oc-cre) will not lead to MPN formation (Dong et al., 2016), which indicates that SHP2 mutations in the specific components of bone marrow microenvironment show leukemogenic effects (Figure 3).

#### SHP2 in Immune Microenvironment Promotes Immune Escape of Tumor Cells

**FIGURE 4:**
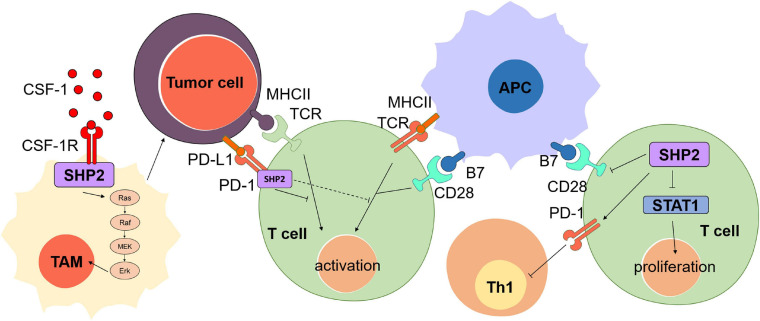
The oncogenic role of SHP2 in tumor immune microenvironment. SHP2 binds to the T cell receptor (TCR) and prevents the activation of T cell induced by the signaling transduction among tumor cells, antigen-presenting cells (APCs), and T cells. SHP2 binds to the colony-stimulating factor-1 receptor (CSF-1R) under the stimulation of CSF-1 and then consequently activates Ras/Raf/MEK/Erk signaling and promotes the proliferation of tumor-associated macrophage (TAM), thus boosting the cancer cell survival. SHP2 in T cells inhibits the activation of T cell via blockage of the function of CD28 and STAT1.

Gain-of-function mutations of SHP2 in tumor microenvironment cells affect tumor progression by non-autonomous mechanisms. SHP2 regulates immune cell functions in the tumor immune microenvironment to affect tumor progressions (Liu et al., 2020; Figure 4). For example, SHP2 regulates the function of T cells by binding to programmed cell death 1 (PD-1) (Liu et al., 2020). PD-1, a key immune checkpoint target for cancer immunotherapy and negative costimulatory receptor, is important to inhibit T cell activation. PD-1 binds to ligand PD-L1 and clusters with T cell receptor (TCR), which is temporarily related to phosphatase SHP2. These negative costimulatory clusters induce dephosphorylation of TCR signal molecules and inhibit the activation of T cells to block TCR induced stop signal (Yokosuka et al., 2012). Dimeric PD-1 activates SHP2-mediated immunosuppression by binding to SH2 domains of SHP2 (N-SH2 and C-SH2) via the C-terminal tyrosine-based switch motif (ITSM) of immune receptor (Okazaki et al., 2001; Sheppard et al., 2004; Yokosuka et al., 2012), thus promoting the immune escape of tumor cells. Other studies reported that cytotoxic T lymphocyte-associated antigen 4 (CTLA-4) is also an immune checkpoint and a negative regulator of T cell immune function (Buchbinder and Desai, 2016; Rowshanravan et al., 2018). Phosphorylation of YYKM motif in CTLA-4 cytoplasmic tail recruits SHP2 to dephosphorylate and inactivate CD28 (Salmond and Alexander, 2006; Lorenz, 2009; Rudd et al., 2009) and to promote the tumor cell survival. Furthermore, SHP2 regulates another signaling to impair the antitumor immunotherapy. SHP2 in the cytoplasm dephosphorylates STAT1, which ultimately inhibits the proliferation of T lymphocytes, leads to a decline in antitumor immunity, and promotes the development of cancer (Liu et al., 2020). Li et al. (2015) analyzed tumor-infiltrating and peripheral blood lymphocytes in head and neck squamous cell carcinoma patients and concluded on the inhibitory effect of SHP2-mediated PD-1 on tumor Th1 cell immunity and that the PD-1 or SHP2 blockade was sufficient to restore Th1 immune activity and to activate T cells, thus reversing immunosuppression in tumor microenvironment.

SHP2 is involved in multiple signaling pathways in tumor-associated macrophages. Stimulated by the colony-stimulating factor-1 (CSF-1), SHP2 binds to the CSF receptor (CSF-1R) complex on the inner membrane of tumor-associated macrophage (TAM), leading to the activation of the Ras/Erk signaling pathway in TAM and supporting the survival, proliferation, and migration of tumor cells (Achkova and Maher, 2016). Furthermore, SHP2 in the macrophages is associated with chronic inflammation-related cancers. Recently, Barkal et al. (2019) have shown that tumor-expressed CD24 binds macrophage sialic-acid-binding Ig-like lectin 10 (Siglec-10) to promote tumor avoidance in the tumor microenvironment by recruiting SHP2 to the cytoplasmic tail ITIM motif of Siglec-10. Besides, Xiao et al. (2019) found that SHP2 deficiency in macrophages disrupts the IL-10/STAT3 signaling pathway, worsening the colons of mice.

### Gain-of-Function SHP2 Promotes Chemoresistance in Cell-Autonomous and/or Non-autonomous Mechanisms

SHP2 promotes chemoresistance in cell-autonomous and/or non-autonomous mechanisms. A previous study observed high expression of SHP2 in both chemoresistant hepatocellular carcinomas (HCCs) and recurrent HCCs derived from patients (Xiang et al., 2017), suggesting a relationship between aberrant SHP2 and chemoresistance. In fact, numerous studies reported that tumor cell-autonomous SHP2 participates in multiple signaling that promotes chemoresistance. For example, SHP2 mutations in tumor cells induce Erk inhibitor resistance through feedback activation of receptor tyrosine kinase (RTK) signaling and rebounding of Erk activity in Erk-driven tumors (Ahmed et al., 2019). SHP2 activates several different tyrosine kinases to drive anaplastic lymphoma kinase (ALK) inhibitor resistance during chemotherapy of ALK-rearranged NSCLCs (Dardaei et al., 2018). SHP2 mediates cisplatin resistance by inhibiting apoptosis and activating the Ras/PI3K/Akt/survivin pathway in lung cancer cells (Tang et al., 2018). Other studies demonstrated that SHP2 activation mutation confers resistance to imatinib in drug-tolerant chronic myeloid leukemia cells. The blockage of Raf/MEK/Erk and PI3K/Akt/mTOR pathways via SHP2 inhibition leads to apoptosis of drug-resistant cells (Li et al., 2018). In PTEN-null senescent tumors, there is a downregulation of SHP2 and activation of JAK/STAT3 pathway, which contributes to the establishment of an immunosuppressive tumor microenvironment that promotes chemoresistance (Toso et al., 2014). SHP2 also influences cancer resistance through other mechanisms. SHP2^*E*76*K*^ activation mutation in bone marrow mesenchymal stromal cells (BMSCs) upregulates vascular cell adhesion molecule 1 (VCAM-1) expression by increasing the PI3K/Akt phosphorylation level and further induces BMSC-mediated chemoresistance in B-cell acute lymphoblastic leukemia (B-ALL) (Yu et al., 2020). This is a typical instance of SHP2 promoting drug resistance in non-autonomous mechanism. In general, these findings illuminate a pivotal oncogenic function of SHP2 in cancers; thus, pharmacological inhibition of SHP2 is a valid therapeutic approach for the treatment of cancers.

## SHP2 Inhibition Is a Promising Antitumor Strategy

### Small-Molecule Inhibitors of SHP2

SHP2 is a potential target for cancer therapy. At present, several SHP2 small-molecule inhibitors are available (Table 2). One study reported an allosteric small-molecule SHP2 inhibitor SHP099, which binds to a tunnel-like pocket formed by the confluence of three domains of SHP2 to stabilize its self-inhibiting conformation, while it has no significant activity against other PTP families (including SHP1) and kinases (Fodor et al., 2018). In addition, SHP099 inhibits the proliferation of RTK-driven human cancer cells by inhibiting Ras/Erk signaling, so drug inhibition of SHP2 is one of the effective strategies for cancer treatment (Chen et al., 2016). Meanwhile, Chen et al. identified a weak SHP2 inhibitor SHP244. X-ray crystallography shows that SHP244 binds to SHP2 and stabilizes the inactive closed conformation of SHP2 by forming cracks at the N-terminal interface between SH2 and PTP (Fodor et al., 2018). In addition, it is possible that the allosteric sites are occupied by SHP099 and SHP244 at the same time, and the combination of SHP099 and SHP244 enhances the pharmacological inhibition of cells (Fodor et al., 2018). A recent study demonstrated that mutated SHP2-mediated LLPS formation is inhibited by SHP2 allosteric inhibitors, which prevent SHP2 from releasing the self-inhibition status. Therefore, the application of SHP2 inhibitors is a promising therapeutic strategy to treat SHP2-involved developmental disorders and tumors (Zhu et al., 2020).

**TABLE 2:** SHP2 small-molecule inhibitors.

SHP2 inhibitors	Characteristics	References
SHP099	Allosteric small-molecule inhibitor, which binds to a tunnel-like pocket formed by the confluence of three domains of SHP2	Chen et al., 2016; Fodor et al., 2018
SHP244	Allosteric small-molecule inhibitor, which binds and stabilizes the inactive, closed conformation of SHP2	Fodor et al., 2018
SHP389	Allosteric small-molecule inhibitor, which binds to a tunnel-like pocket formed by the confluence of three domains of SHP2 and modulates MAPK signaling *in vivo*	Bagdanoff et al., 2019
SHP394	Allosteric small-molecule inhibitor, an orally efficacious inhibitor of SHP2, with high lipophilic efficiency, improved potency, and enhanced pharmacokinetic properties	Sarver et al., 2019
MRC-4550	Allosteric small-molecule inhibitor, which targets phosphatase activity of SHP2	Nichols et al., 2018
RMC-4630	Allotropic selective inhibitor, which is being evaluated in a multi-cohort phase I/II clinical program	Moore et al., 2020
PCC0208023	Allosteric small-molecule inhibitor, which shows higher affinity with key residues in the SHP2 allosteric pocket	Chen et al., 2020
NSC-87877	Binds to the catalytic cracking of SHP1/2 PTP and inhibits EGF-induced Erk1/2 activation *in vitro*	Song et al., 2009; Shi et al., 2015
PHPS1	Effective cell permeation inhibitor, which shows efficacy in blocking the downstream signal pathway dependent on SHP2	Chen et al., 2018; Salem et al., 2018
Cefsulodin	Blocks SHP2-mediated signal transduction and proliferation of several cancer cell *in vitro*	He et al., 2015

Recently, Bagdanoff et al. (2019) identified SHP2 inhibitor SHP389, which regulates MAPK signal *in vivo*. Another study improved the basis of the allosteric inhibitors described previously and identified a new effective oral SHP2 inhibitor, SHP394, which shows high lipid efficiency, improved efficacy, and enhanced pharmacokinetics (Sarver et al., 2019). Nichols et al. (2018) showed that MRC-4550 affects human tumor models. MRC-4550 treatment reduces Ras/Raf/MEK/Erk signal transduction and cancer growth. RMC-4630 is an oral and effective allotropic selective inhibitor of SHP2 in the Ras signaling pathway. This inhibitor is currently in clinical trials and is being evaluated in a multi-cohort phase 1/2 clinical program (Moore et al., 2020). An effective SHP2 variable structure allosteric inhibitor PCC0208023 was synthesized recently. It non-competitively inhibits the activity of SHP2. In addition, PCC0208023 inhibits the proliferation of human CAC cells driven by KRAS mutation by inhibiting Ras/MAPK signaling pathway *in vitro*. It also shows an antitumor effect on KRAS-driven xenograft model (Chen et al., 2020). Other studies have identified NSC-87877, which binds to the catalytic cracking of SHP2 PTP (Chen et al., 2006). Additionally, PHPS1 is an effective cell permeation inhibitor, which inhibits SHP2-dependent cell processes, such as hepatocyte growth factor/dispersant factor (HGF/SF)-induced epithelial cell scattering and branching. PHPS1 also blocks the SHP2-associated downstream signaling pathway, such that it inhibits the SHP2^*E*76*K*^-mediated activation of Erk1/2 to prevent the growth of a variety of human tumor cell lines (Hellmuth et al., 2008). Wang et al. found that cefsulodin blocks SHP2-mediated signaling transduction and proliferation of several cancer cell lines (He et al., 2015).

### Small-Molecule Inhibition of Cell-Autonomous SHP2 to Prevent Chemoresistance

SHP2 is a potential therapeutic target for cancer treatment, as it plays a significant role in promoting chemoresistance. Chemotherapy is one of the most commonly used methods in the clinical diagnosis and treatment of malignant tumors. It is an effective means of systemic treatment for not only the tumors at the treatment site but also the clinical metastasis tumors at the potential lesions (Arbour and Riely, 2019). However, the resistance of tumor cells to chemotherapy drugs often impairs its efficacy and finally leads to failure, which has become a huge challenge for cancer treatment (Vasan et al., 2019). Increasing evidences have shown that mutated SHP2 plays an important role in chemoresistance (Ruess et al., 2018). At present, multiple allosteric inhibitors of SHP2 are discovered (Chen et al., 2016), and traditional chemotherapeutic drugs combined with SHP2 inhibition have become a potential approach to enhance efficacy of chemotherapy and immunotherapy.

**FIGURE 5:**
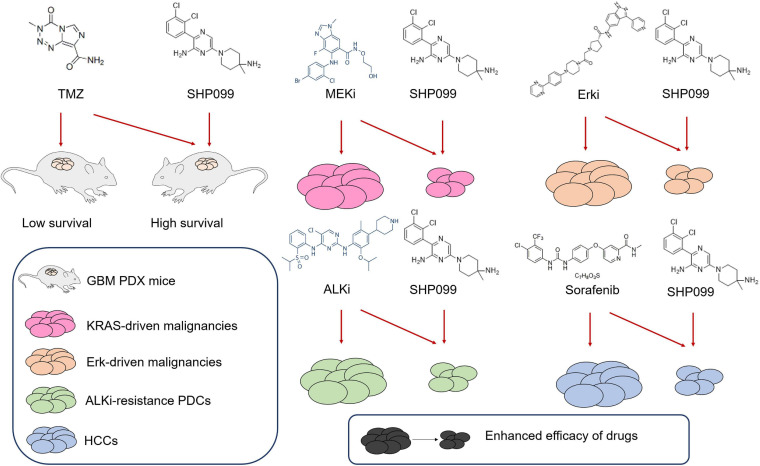
Combination of SHP099 with other drugs enhances antitumor efficacy. In patient-derived xenograft (PDX) mouse model, temozolomide (TMZ) combines with SHP099 to increase the survival rate of tumor-carrying mice. The combination of SHP099 with MEK inhibitor, Erk inhibitor, ALK inhibitor, or sorafenib impairs the survival of malignancies, which indicates that the drug toxicity is enhanced through the drug combination.

Some studies reported that the combination of SHP2 inhibitors with other drugs shows promising application prospects (Figure 5). SHP099 exhibited antitumor activity either as a single agent or in combination with temozolomide (TMZ) and provided significant survival benefits for GBM tumor xenograft-bearing animals (Sang et al., 2019). MEK inhibitors show limited efficacy, because of the rapid development of adaptive resistance, whereas SHP2 inhibitor SHP099 combined with MEK inhibition prevents adaptive resistance in multiple KRAS-driven malignancies (Fedele et al., 2018). Pharmacologically targeting Erk signaling in Erk-dependent tumors is also limited by adaptive resistance, due to the feedback activation of RTK signaling, which is mediated by SHP2. Thus, targeting Erk signaling and SHP2 prevents such resistance in Erk-dependent tumors (Ahmed et al., 2019). Most ALK-rearranged NSCLCs initially respond to small-molecule ALK inhibitors, but drug resistance often develops. Researchers identified SHP2 as a common targetable resistance node in multiple ALK inhibitor-resistance patient-derived cells (PDCs), and treatment with SHP099 in combination with the ALK TKI ceritinib blocked the growth of resistant PDCs by preventing compensatory Ras and Erk reactivation (Dardaei et al., 2018). The survival benefit of sorafenib for patients with HCC is unsatisfactory due to the development of adaptive resistance, and SHP2 was observably upregulated in sorafenib-resistant HCC cell lines as well as patient-derived xenografts (Leung et al., 2019). SHP2 inhibition by SHP099 in combination with sorafenib attenuated the adaptive resistance to sorafenib by impeding RTK-induced reactivation of the MEK/Erk and Akt signaling pathways. Dioscin inhibits MEK/Erk and PI3K/Akt signaling pathways to abrogate TKI resistance through dysregulation of SHP2 expression in lung adenocarcinoma (Wang Y.C. et al., 2018). Thus, the combination of dioscin and TKI is potentially therapeutic for chemoresistant tumor treatment.

### SHP2 Inhibition Is a Potential Strategy for Immunotherapy

SHP2 inhibition is a potential strategy for enhancing the efficacy of antitumor immunotherapy. Pharmacological inhibition of SHP2 through SHP099 combined with PD-1 antibody is a valid therapeutic approach for the treatment of cancers through enhancing the efficacy of antitumor immunity (Zhao M. et al., 2019). Some preclinical findings revealed that SHP2 promotes immune suppression in the tumor microenvironment; thus, the allosteric inhibition of SHP2 by RMC-4550 could induce antitumor immunity (Quintana et al., 2020). Other studies demonstrated that the co-inhibition of CSF1-R and SHP2 using nanoparticles loaded with inhibitors for tumor TAM activation and enhancement of phagocytosis is an effective strategy for macrophage-based antitumor immunotherapy (Ramesh et al., 2019). A recent study reported that allosteric inhibition of SHP2 leads to direct and selective depletion of pro-tumorigenic M2 macrophages and promotes antitumor immunity, suggesting a therapeutic approach for Ras-driven cancers (Quintana et al., 2020).

## Conclusion and Discussion

SHP2 serves as a pivotal hub to connect multiple oncogenic signaling pathways, such as PI3K/Akt, Ras/Raf/MAPK, and PD-1/PD-L1 pathways. It promotes tumor progression via cell-autonomous and non-cell-autonomous mechanisms. That is, on the one hand, activation mutations of SHP2 in specific cells directly establish tumorigenic phenotype to promote the tumor progressions; on the other hand, SHP2 mutations in the tumor microenvironment promote tumor development. Oncogenic SHP2 is regarded as a potential cancer treatment target. Recently, multiple types of SHP2 inhibitors have been discovered to enhance cancer treatments.

At present, there are several techniques to study gene functions and explore new antitumor targets. Except for the Cre/LoxP system, clustered regularly interspaced short palindromic repeats (CRISPR)/Cas9-based genome-wide screening is widely applied to study the gene functions and to discover novel targets for treatment. CRISPR/Cas9 is a gene-editing tool for operating specific genes in the genome, which was first found as part of the adaptive immune system in bacteria. In recent years, CRISPR/Cas9 has been widely applied in altering genomes to activate or to repress the expression of genes; thus, its application accelerates the study of the mechanism of tumorigenesis and the development of cancer therapy (Lee et al., 2019). A recent study demonstrated that genome-scale CRISPR/Cas9 gene-knockout screening is applied in discovering potential therapeutic antitumor targets in the cancer cells’ genomes and identified the important role of *PTPN11* in pediatric rhabdoid tumors (Oberlick et al., 2019). Genome-scale CRISPR/Cas9 gene-knockout screening can also be applied to study protein function and to explore new therapeutic targets. Recent studies applied the CRISPR/Cas9 system to gene therapy. AAV-CRISPR/Cas9-mediated gene editing corrects *Ldlr* mutation *in vivo* and effectively ameliorates atherosclerosis phenotypes, which is a potential therapeutic approach for patients with familial hypercholesterolemia (Zhao et al., 2020). Since transfusing the PD-1 knockout T-cells to patients with solid tumor induces immunological responses against tumor cells, the CRISPR/Cas9 system is regarded as therapeutic tool (Zhan et al., 2019). With the development of drug delivery systems (Chen et al., 2017; Wang P. et al., 2018), whether CRISPR/Cas9 could be applied to edit SHP2 mutation for enhancement of cancer therapy is worth exploring.

Other targeting SHP2 degradation techniques are also potential adjuvant approaches for enhancement of cancer therapeutic efficacy, including proteolysis-targeting chimera (PROTAC). PROTAC is designed to allosterically target specific proteins and recruit the E3 ligase Von Hippel-Lindau (VHL), resulting in ubiquitination and subsequent degradation of the target protein (Burslem et al., 2019). This technique is widely applied to drug development and research on mechanisms of chemoresistance. To date, about 50 proteins, including clinically validated drug targets, are targeted by PROTAC for degradation, and these PROTACs have been successfully developed in clinical trials for cancer therapy (Li and Song, 2020). For example, PROTAC-induced bromodomain and extra-terminal (BET) protein degradation showed anti-prostate cancer efficacy (Raina et al., 2016). A recent study demonstrated that induced SHP2 degradation through PROTAC is an effective approach to inhibit the function of SHP2, and it further pointed out that optimization of these SHP2 degraders may lead to the development of a new class of therapies for cancers and other human diseases (Wang et al., 2020).

Recently, hyperactivation of SHP2 through the formation of LLPS has been elucidated (Zhu et al., 2020). Both GOF and LOF disease-associated SHP2 variants promote LLPS to increase the catalytic activity of mutant and wild-type SHP2, leading to MAPK hyperactivation. SHP2 is an important signal hub in normal conditions, and its hyperactivation will undoubtedly lead to the breakdown of cell signal balance. Therefore, in developmental diseases, especially tumors, LLPS possibly acts as an important driver for disease occurrence and chemotherapeutic resistance. As it has been found that allosteric inhibitors of SHP2 have an inhibitory effect on the formation of LLPS, it is very promising to develop new therapies based on SHP2 inhibition and LLPS blockage.

## Author Contributions

LD and DH drafted the manuscript and designed the structure. QX, MX, and XM proposed useful comments, suggestions, and revised the manuscript. CZ revised the language of the manuscript. All authors contributed to the article and approved the submitted version.

## Conflict of Interest

The authors declare that the research was conducted in the absence of any commercial or financial relationships that could be construed as a potential conflict of interest.
